# VEXAS syndrome caused by a *UBA1* mutation is complicated by recurrent infections leading to hemophagocytic lymphohistiocytosis

**DOI:** 10.1016/j.gendis.2025.101540

**Published:** 2025-01-22

**Authors:** Yu Tang, Hongfei Cui, Hongjun Zhao, Hui Luo, Xiaoxia Zuo, Junjiao Wu

**Affiliations:** Aging Research Center, Department of Geriatrics, Xiangya Hospital, Central South University, Changsha, Hunan 410008, China; National Clinical Research Center for Geriatric Disorders, Xiangya Hospital, Central South University, Changsha, Hunan 410008, China; National Clinical Research Center for Geriatric Disorders, Xiangya Hospital, Central South University, Changsha, Hunan 410008, China; Department of Rheumatology and Immunology, Xiangya Hospital, Central South University, Changsha, Hunan 410008, China; Provincial Clinical Research Center for Rheumatic and Immunologic Diseases, Xiangya Hospital, Central South University, Changsha, Hunan 410008, China; Hunan Provincial Skin Immunization and Medical Center, Changsha, Hunan 410008, China

VEXAS syndrome (vacuoles, E1 enzyme, X-linked, autoinflammatory, somatic) is an adult-onset and treatment-refractory inflammatory disease, caused by acquired mutations in *UBA1* (ubiquitin-like modifier activating enzyme 1), an X-linked gene encoding an E1 enzyme of the ubiquitin-proteasome system.^1^ The disease occurs predominantly in elderly male patients and has heterogeneous but expanding clinical features that include fever, characteristic vacuoles in hematopoietic precursors, cytopenias, and chondritis. As such, VEXAS patients may be initially diagnosed with relapsing polychondritis, myelodysplastic syndrome, and other syndromes.

Here, we report the first Chinese VEXAS patient carrying a hemizygous NM_003334.4:c.121A > G (p.M41V) *UBA1* mutation (Fig. 1A), detected by a whole-exome sequencing analysis at a variant allele frequency of 69.6%, one of the three major *UBA1* mutations occurring in VEXAS syndrome. The patient was a 50-year-old man who initially presented with long-lasting recurrent fever, auricular chondritis, and maculopapular rash, without neurological manifestations (Fig. 1B, C). The patient was hospitalized three times from Nov 25, 2022, to Apr 30, 2024, in our hospital. Essentially, the patient's red blood cell counts, hemoglobin levels, and platelet levels were consistently far below normal ranges, dropping down rapidly, especially upon a new infection (Fig. 1D–G and Table S1). The positron emission tomography/computed tomography scan revealed diffuse elevation of bone marrow glucose metabolism throughout the body (Fig. S1), reminiscent of bone marrow activation. Inflammatory markers, including C-reactive protein and erythrocyte sedimentation rate, were consistently increased (Table S1). Moreover, serum cytokines such as interferon (IFN)-γ, interleukin (IL)-6, IL-8, and IL-17 were substantially elevated (Table S2). The patient had aberrant immune responses and recurrent infections, as neutrophil ratios were mostly increased (Fig. 1H–K). However, lymphocyte ratios, assessed by TBNK assays, as well as natural killer cell activities were all normal (Table S3). Immunoglobin (IgA, IgG, IgM), autoimmune antibodies (antinuclear antibodies and antiplatelet antibodies), and complements (C3, C4) were also normal.

**Figure 1:**
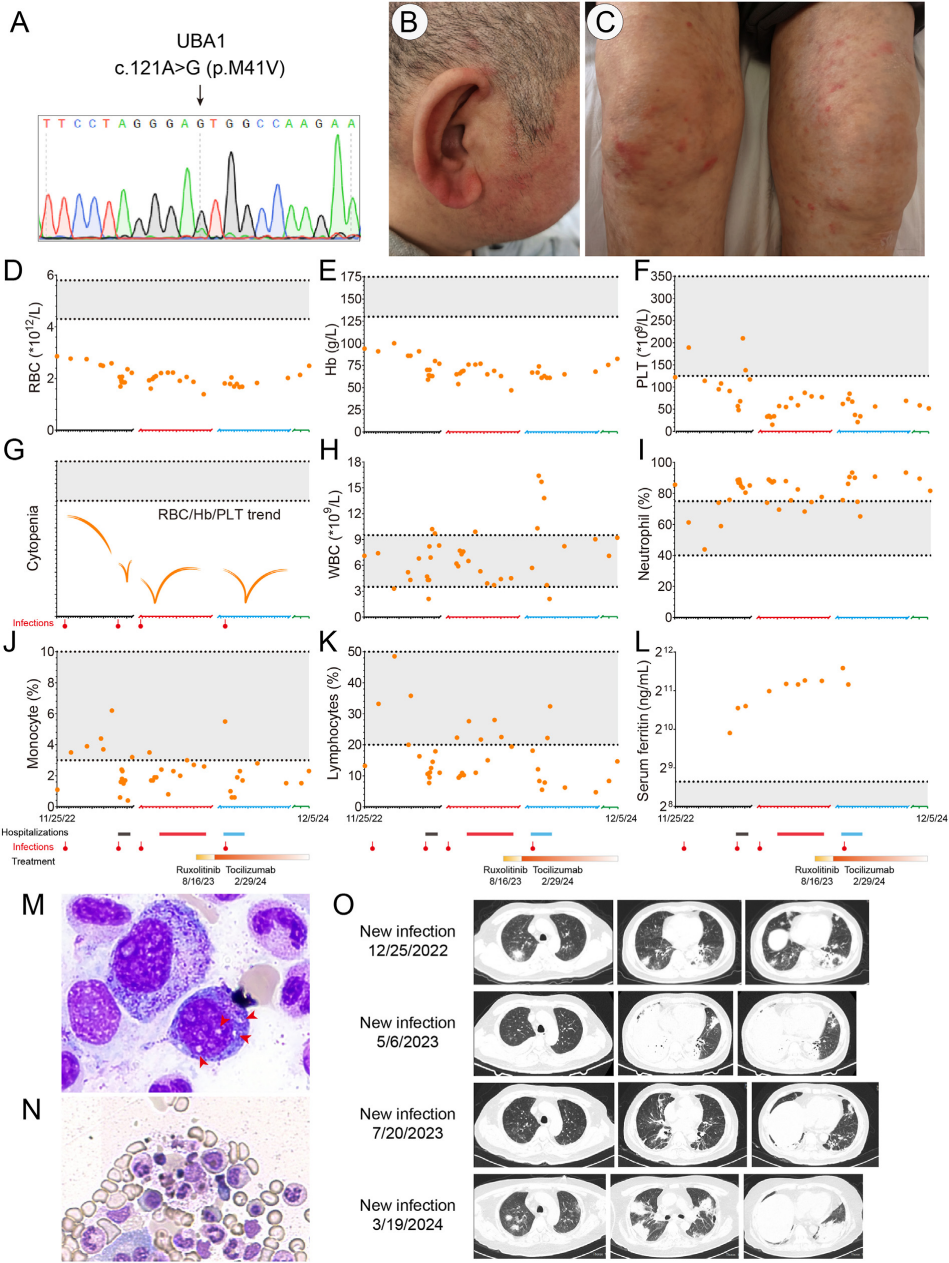
Molecular and pathological manifestations of this VEXAS case. **(A)** Sanger sequencing of the peripheral blood sample showed a mosaic mutation of *UBA1* gene (c.121A > G; p.M41V). **(B, C)** The patient was a 50-year-old man who initially presented with lasting fever, auricular chondritis, and maculopapular rash on the trunk and extremities. **(D**–**K)** The patient was hospitalized three times from Nov 25, 2022, to Apr 30, 2024, in our hospital. Essentially, the patient's red blood cell (RBC) counts, hemoglobin (Hb) levels, and platelet (PLT) levels were consistently far below normal ranges, dropping down rapidly, especially upon a new infection (G). The patient had aberrant immune responses and recurrent infections, as neutrophil ratios were mostly increased whereas both monocyte and lymphocyte ratios were relatively decreased. The dash lines indicate normal range thresholds. WBC, white blood cell. Hospitalization periods were labeled with colored boxes: black box: 05/05/2023∼06/02/2023; red box: 07/27/2023∼08/22/2023; blue box: 03/13/2024∼03/28/2024. Infections (with red labels) were recorded on 12/25/2022, 05/06/2023, 07/20/2023, and 03/19/2024, respectively. The *X*-axis indicates date ranges: black axis: 11/28/2022∼06/09/2023; red axis: 07/15/2023∼08/25/2023; blue axis: 03/10/2024∼05/01/2024; green axis: 11/14/2024 and 12/05/2024. **(L)** The levels of serum ferritin were consistently far above normal ranges. **(M, N)** Bone marrow aspiration revealed characteristic cytoplasmic vacuoles (red arrowheads) in myeloid precursors and prominent hemophagocytosis by Wright-Giemsa staining. **(O)** Chest computed tomography scans showed an intermittent course of new infection and absorption of the VEXAS patient, as assessed by typical pulmonary consolidations and ground-glass opacities in his lungs.

Bone marrow biopsy revealed characteristic cytoplasmic vacuoles in myeloid precursors, a typical hallmark for VEXAS syndrome (Fig. 1M), and prominent hemophagocytosis (Fig. 1N). Moreover, both flow cytometry analysis of bone marrow lymphocytes and chromosomal karyotype analysis did not show any obvious abnormalities, which suggests no evidence of myelodysplastic syndrome. Interestingly, serum ferritin and sCD25 (soluble cluster of differentiation 25)/IL-2R levels were consistently elevated (Fig. 1L and Table S4). Taken together, the patient's symptoms met at least five out of the eight hemophagocytic lymphohistiocytosis (HLH)-2004 diagnostic criteria, including long-lasting fever, cytopenia (decreased in the erythroid and megakaryocytic lineages), hemophagocytosis, hyperferritinemia, and elevated sCD25/IL-2R (Table S4).

Chest computed tomography scans showed an intermittent course of new infection and absorption. Typically, computed tomography images manifested multiple flaky, ground-glass opacities and pulmonary consolidations (Fig. 1O). Next-generation sequencing tests of bronchoalveolar lavage fluid identified a variety of pathogens such as *SARS-CoV-2*, *Rickettsia*, *Haemophilus parainfluenzae*, *Streptococcus mitis*, and *Malassezia furfur* (Table S4). Tuberculosis, however, was not detected by IFN-γ release assays (Table S5). In addition, *cytomegalovirus* and *Epstein–Barr virus* were inactivated, albeit with moderately increased loads (Table S6).

Although the patient had been continuously treated with anti-bacterial and anti-fungal drugs, the inflammatory symptoms (such as fever and cytokine storm) were remarkably ameliorated only with prednisone, ruxolitinib, or tocilizumab treatment, since Aug. 2023. Specifically, ruxolitinib treatment improved the inflammation state and decreased the frequency of fever. Five days after ruxolitinib treatment (08/21/2023), serum cytokines such as IFN-α, IFN-γ, IL-2, IL-5, IL-8, IL-12p70, and IL-17 were remarkably reduced (Table S2). However, the patient still suffered recurred fever occasionally before Nov. 2023 when VEXAS syndrome was diagnosed, and methylprednisone (40 mg/d) was given.

Notably, ruxolitinib was discontinued (since Feb. 2024) due to worsened anemia. After that, tocilizumab (480 mg/3–4 weeks) and low-dose prednisone (10 mg/d and tapering to 7.5 mg/d) were regularly given. After treatment with tocilizumab for 14 days (03/14/2024), tests of serum cytokines showed that IL-6 and IFN-γ level was 298.25 pg/mL and 130.82 pg/mL, respectively, while levels of other cytokines such as IFN-α, IL-2, IL-4, IL-5, IL-8, IL-12p70, and IL-17 were all decreased back to normal ranges (Table S2).

The patient was then followed up for 8 months until Dec. 2024, during which there were no instances of fever. Before injection of tocilizumab every 3–4 weeks, the patient may experience mild muscle aches/pains or a few new rashes, along with elevated levels of C-reactive protein (Table S1). However, all these symptoms could be alleviated after the administration of tocilizumab. Basically, the hemoglobin level was maintained at around 70.0–80.0 g/L, and the platelet level was maintained at around 60.0 × 10^9^/L (Fig. 1). The coagulation examination was relatively normal (Table S7). As such, the patient was in a stable condition that greatly improved his life quality. A comprehensive medical history, including detailed pathological manifestations and treatments, is described in the supplementary file and is briefly summarized in a chronicle in Figure S2.

Not surprisingly, VEXAS syndrome presents a significant predisposition to infections, a consequence of acquired immunodeficiency stemming from a compromised ubiquitin-proteasome system as well as immunosuppressive therapies.^2^^,^^3^ Within a French VEXAS registry,^3^ infections were prevalent. Elderly patients (>75 years) carrying the p.M41V mutation and treated with JAK inhibitors exhibited an increased incidence of serious infections.^3^ So far, several cases of VEXAS syndrome complicating HLH have been documented, all presenting with infections by multiple pathogens (summarized in Table S4).

The causal relationship between VEXAS and HLH, however, remains elusive. Here, we propose that the hyperimmune milieu, caused by *UBA1* mutations, presents an intrinsic risk for HLH, while susceptibility to infections acts as a second hit. This hypothesis aligns with our current understanding of HLH, which often involves a combination of genetic predisposition and triggering factors like infections or malignancies. To support this, first, we identified no HLH or myeloid-associated genetic variants in this patient after whole-exome sequencing detection, excluding the possibility of familial HLH (Table S8). Second, in this study, the patient's cytopenia worsened rapidly upon each new infection (Fig. 1G), suggesting that infections are highly possible to give a second hit to developing HLH in VEXAS patients. However, due to the rarity of VEXAS, further studies are crucial to validate this.

To date, there are only two documented cases of VEXAS syndrome in China with distinct *UBA1* mutations (p.M41T and p.M41L).^4^^,^^5^ This study presents the first Chinese VEXAS patient carrying a p.M41V mutation, which is linked with a more severe phenotype due to lower residual translation of the normal cytoplasmic UBA1 isoform UBA1b.^1^ Given the recurrent infections and HLH symptoms in this patient, it is imperative to closely monitor disease progression and potential drug resistance. The increased incidence of infections associated with the p.M41V mutation^3^ also underscores the importance of high suspicion for opportunistic infections before intensifying immunosuppressive treatment, while also implementing anti-infective prophylaxis in patient care.

Overall, this study provides both clinicians and researchers with a deeper understanding of the causal relationship between the genetic basis of VEXAS and recurrent infection-associated HLH and has important implications for exploring its significance in diagnosis, treatment, and patient care.

## Ethics declaration

All participants provided written informed consent, which is approved by the Institutional Review Board of the Xiangya Hospital of Central South University (No. 202405007) and abides by the Declaration of Helsinki principles. Written informed consents were obtained by the patient and his family members.

## Funding

This study was funded by the National Natural Sciences Foundation of China (No. 82271280 to Y.T.; 82301433 to J.J.W.), 10.13039/501100004735Hunan Provincial Natural Science Foundation of China (No. 2022JJ40824 to J.J.W.), Scientific Research Project of Hunan Provincial Health Commission (China) (No. B202303070054 to Y.T.), Talents Startup Fund (China) (No. 2209090550 to Y.T.), Youth Science Fund (China) (No. 2021Q04 to J.J.W.), and the Project Program of National Clinical Research Center for Geriatric Disorders of 10.13039/501100011790Xiangya Hospital, Central South University, Changsha, China (No. 2022LNJJ14 to H.J.Z.).

## CRediT authorship contribution statement

**Yu Tang:** Writing – review & editing, Writing – original draft, Funding acquisition, Conceptualization. **Hongfei Cui:** Resources, Data curation. **Hongjun Zhao:** Resources, Data curation. **Hui Luo:** Resources, Data curation. **Xiaoxia Zuo:** Data curation, Resources. **Junjiao Wu:** Writing – review & editing, Writing – original draft, Investigation, Funding acquisition, Conceptualization.

## Data availability

The data were available upon request from the corresponding author.

## Conflict of interests

The authors have declared that no competing interest exists.
