# Supplementary material for: VEXAS syndrome caused by a UBA1 mutation is complicated by recurrent infections leading to hemophagocytic lymphohistiocytosis
Source: Genes Dis. 2025 Jan 22;12(5):101540. doi: 10.1016/j.gendis.2025.101540 (PMC12099774; doi:10.1016/j.gendis.2025.101540)
Supplement: Multimedia component 1 [file mmc1.docx]

**Supplementary files**

**Supplementary Figure 1. PET/CT of the VEXAS patient.** The PET/CT scan revealed diffuse elevation of bone marrow glucose metabolism throughout the body, reminiscent of bone marrow activation.

**
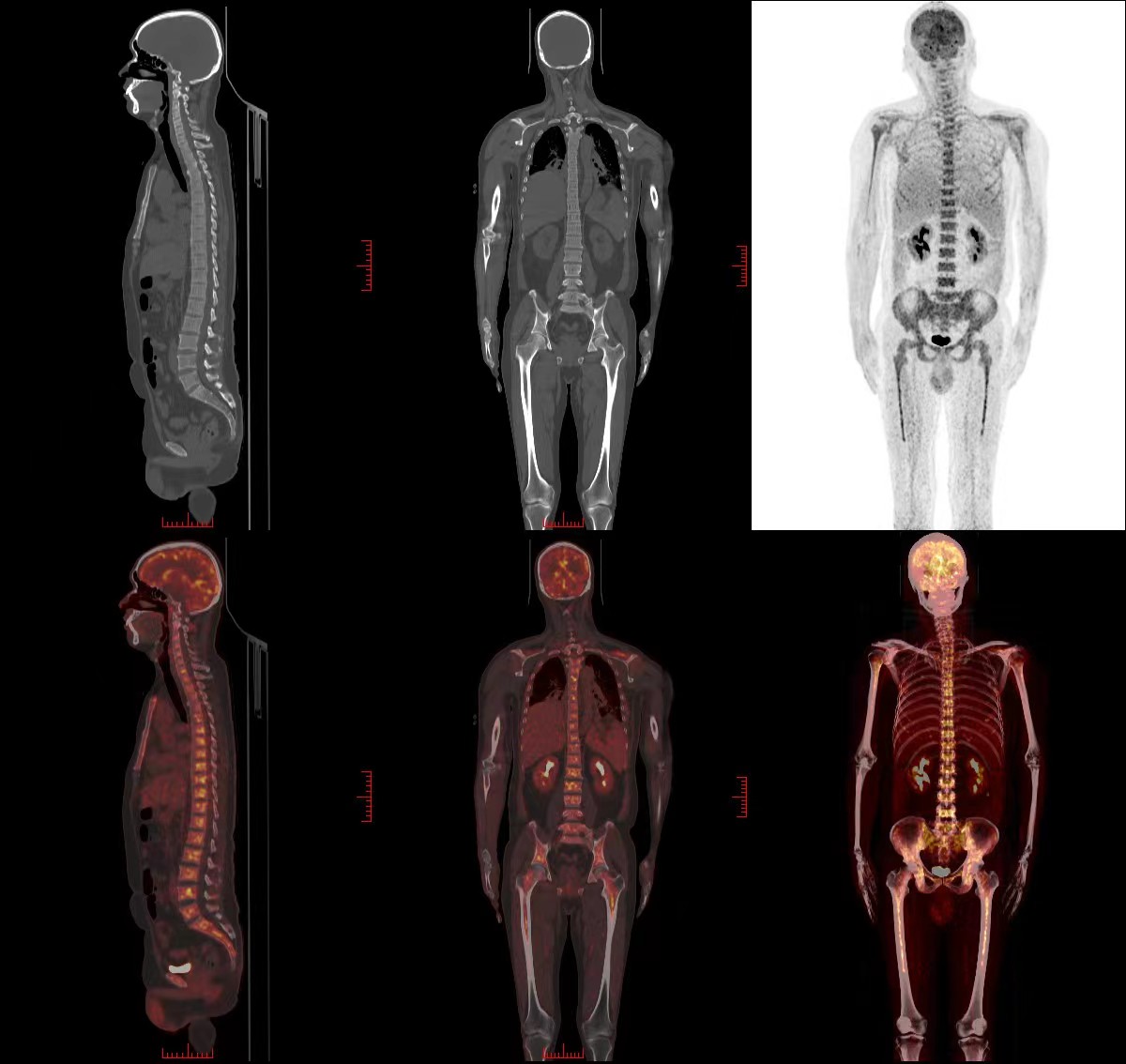
**

**Supplementary Figure 2. Summary of the medical history of the VEXAS patient**

**
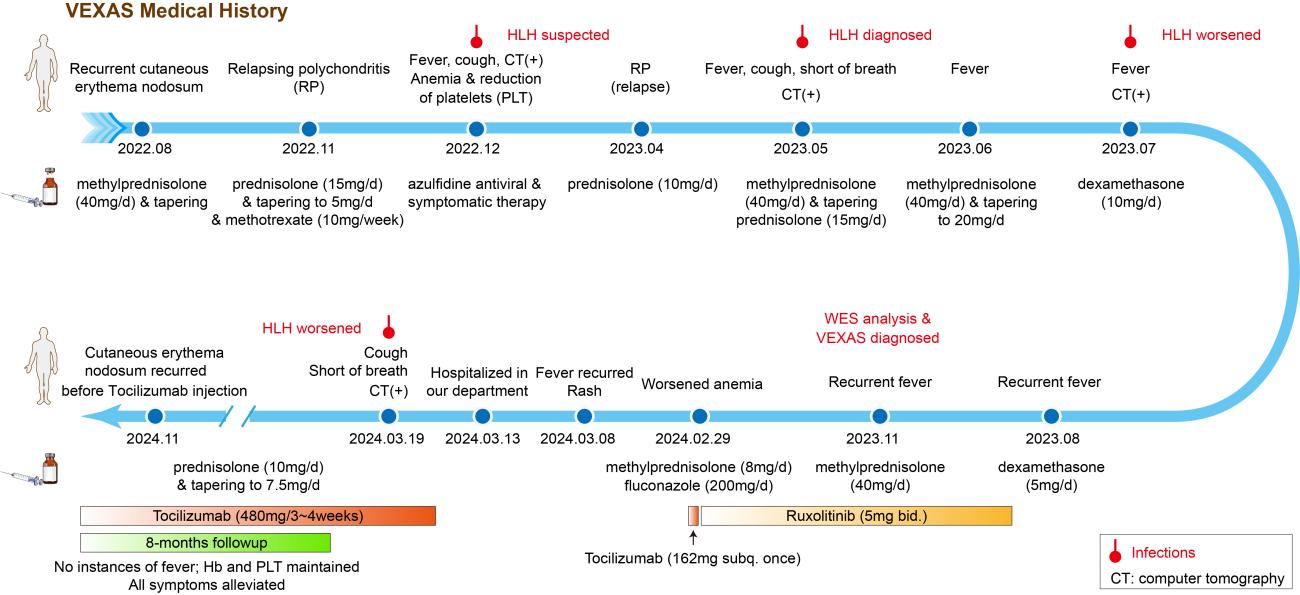
**

**Supplementary Table 1. Laboratory tests of the VEXAS patient**

|  |  |  | MCV  (fl) | Ret (10^9^/L) | CRP (mg/L) | ESR (mm/h) | TP (g/L) | ALb (g/L) | AST (U/L) | LDH (U/L) | PCT (ng/mL) | DD (mg/L) | TG (mM) | FIB  (g/L) |
| --- | --- | --- | --- | --- | --- | --- | --- | --- | --- | --- | --- | --- | --- | --- |
| Hospitalization | Days | Date | [80-100] | [24-84] | [0-6] | [0-21] | [65-85] | [40-55] | [15-40] | [120-250] | [<0.05] | [<0.5] | [<1.7] | [2-4] |
|  | 1 | 12/25/22 | 99.3 |  | 1.48 | 30 | 65 | 32.9 | 10.8 |  |  |  |  |  |
|  | 10 | 01/03/23 | 103.2 |  | 45.7 | 35 | 37.6 | 34.9 | 6.5 | 270 |  |  |  |  |
|  | 51 | 02/13/23 | 106.3 |  | 2.51 | 44 | 29.6 | 43.7 | 4.3 |  |  |  |  |  |
|  | 87 | 03/21/23 | 106.3 |  | 108 | 120 | 29.7 | 41.8 | 3.4 |  |  |  |  |  |
|  | 93 | 03/27/23 | 104.8 | 49.3 |  |  | 29.8 | 43 |  |  |  |  |  |  |
|  | 115 | 04/18/23 | 107.7 |  |  | 120 |  |  |  |  |  |  |  |  |
| 1st | 136 | 05/09/23 | 107.8 |  |  |  | 25.9 | 37.4 | 6.4 | 204 |  |  | 0.84 |  |
|  | 138 | 05/11/23 | 106.3 |  |  |  |  |  |  |  |  | 1.38 |  | 11.3 |
|  | 140 | 05/13/23 | 106.2 |  |  |  |  |  |  |  |  |  |  |  |
|  | 141 | 05/14/23 | 104.7 |  |  |  |  |  |  |  |  |  |  |  |
|  | 142 | 05/15/23 | 101.9 | 18 | 224 | 88 | 24.2 | 26.7 | 10.4 |  | 1.251 | 1.74 |  | 18.7 |
|  | 145 | 05/18/23 | 104.6 |  |  |  |  |  |  |  |  |  |  |  |
|  | 149 | 05/22/23 | 100.6 |  | 91 |  | 30.4 | 32.2 | 10 | 222 | 0.456 | 1.43 |  | 15.1 |
|  | 156 | 05/29/23 | 101.9 |  | 17.2 | 79 | 26.2 | 32 | 10.4 |  | 0.154 | 0.48 |  | 5.9 |
|  | 167 | 06/09/23 | 112 |  |  | 120 | 26.1 | 35.8 | 9.2 |  |  |  |  |  |
| 2nd | 208 | 07/20/23 | 107.8 | 35.3 |  |  | 33.8 | 29.6 | 8.4 |  |  | 0.88 | 1.68 | 10.2 |
|  | 209 | 07/21/23 | 106.2 |  |  | 73 |  |  |  | 252 |  |  |  |  |
|  | 210 | 07/22/23 | 105.6 |  |  |  |  |  |  |  |  |  |  |  |
|  | 211 | 07/23/23 | 102.9 |  |  |  |  |  |  |  |  |  | 0.78 |  |
|  | 212 | 07/24/23 | 104.3 |  |  |  | 31.4 | 30.1 | 4.8 | 194 |  |  |  |  |
|  | 215 | 07/27/23 | 105 | 31.9 |  |  | 27.8 | 35 | 5.8 | 169 | 0.298 | 0.98 | 2.31 | 11.3 |
|  | 219 | 07/31/23 | 106 |  |  |  |  |  |  |  |  |  |  |  |
|  | 222 | 08/03/23 | 106 |  |  |  |  |  |  |  |  |  |  |  |
|  | 226 | 08/07/23 | 106.3 |  |  |  | 23.6 | 30.4 | 5.2 | 204 | 0.303 |  | 1.16 |  |
|  | 230 | 08/11/23 | 104.2 |  |  |  | 27.4 | 32.9 | 6.8 | 230.6 | 0.398 |  | 1.89 |  |
|  | 234 | 08/15/23 | 104 |  |  |  | 26.1 | 32 | 5.5 | 238 | 0.258 |  | 1.6 |  |
|  | 240 | 08/21/23 | 105 |  |  |  | 26.5 | 28.5 | 6.9 | 208.9 | 0.231 |  | 0.81 |  |
| 3rd | 446 | 03/14/24 | 110.1 |  | 109 | 52 | 24.5 | 32.3 | 9.1 | 411 | 0.36 | 0.76 | 1.98 | 7.1 |
|  | 450 | 03/18/24 | 109.4 |  | 13.5 |  | 22.4 | 33.7 | 7.2 |  |  | 0.73 |  |  |
|  | 451 | 03/19/24 | 110.4 |  | 15.6 |  |  |  |  | 392 |  | 0.95 |  | 8 |
|  | 453 | 03/21/24 | 110.6 |  | 31.7 |  |  |  |  | 423 |  |  |  |  |
|  | 455 | 03/23/24 | 114 |  | 6.84 |  |  |  |  |  | 0.61 |  |  |  |
|  | 457 | 03/25/24 | 110.8 |  | 2.96 |  |  |  |  |  |  |  |  |  |
|  | 459 | 03/27/24 | 108.4 |  |  |  | 18.8 | 35 | 7.1 |  | 0.2 |  |  |  |
|  | 470 | 04/07/24 | 106 |  |  |  |  |  |  |  |  |  |  |  |
|  | 493 | 04/30/24 | 107 |  |  |  |  |  |  |  |  |  |  |  |
| Followup | | 11/14/24 | 116 |  | 137.4 | 149 | 60.8 | 37.7 | 5.9 | 177.3 | 0.32 | 0.69 | 1.46 | 6.65 |
|  |  | 12/05/24 | 114 |  | 44.51 | 93 |  |  |  |  |  | 0.88 |  | 5.62 |

MCV, mean corpuscular volume; Ret, reticulocyte; CRP, C-reactive protein; ESR, erythrocyte sedimentation rate; TP, total protein; ALb, albumin; AST, aspartate aminotransferase; LDH, lactate dehydrogenase; PCT, procalcitonin; DD, D-dimer; TG, triglycerides; FIB, fibrinogen

**Supplementary Table 2. Laboratory values of serum cytokine concentrations of the VEXAS patient**

| **Cytokines** | **Value**  **08/15/2023** | **Value**  **08/21/2023 *** | **Value**  **03/14/2024 **** | **Units** | **Reference range** |
| --- | --- | --- | --- | --- | --- |
| IFN-α | 39.88 (↑) | 17.90 | 1.13 | pg/mL | <=16.5 |
| IFN-γ | 189.40 (↑) | 24.70 | 130.82 (↑) | pg/mL | <=23.1 |
| IL-2 | 33.04 (↑) | 15.75 (↑) | 1.41 | pg/mL | <=7.5 |
| IL-4 | 10.03 (↑) | 3.56 | 2.03 | pg/mL | <=8.56 |
| IL-5 | 37.18 (↑) | 7.08 (↑) | 1.79 | pg/mL | <=3.1 |
| IL-6 | 157.26 (↑) | 118.38 (↑) | 298.25 (↑) | pg/mL | <=5.4 |
| IL-8 | 135.12 (↑) | 45.39 (↑) | 1.68 | pg/mL | <=20.6 |
| IL-10 | 11.54 | 4.81 | 6.40 | pg/mL | <=12.9 |
| IL-12p70 | 6.16 (↑) | 3.07 | 1.14 | pg/mL | <=3.4 |
| IL-17 | 49.35 (↑) | 17.90 | 2.28 | pg/mL | <=21.4 |
| TNF-α | 0.65 | 0.65 | 12.50 | pg/mL | <=16.5 |

* 5 days after ruxolitinib treatment; Ruxolitinib was discontinued in Feb 2024 due to worsened anemia

** 14 days after tocilizumab treatment

**Supplementary Table 3. The T-, B- and NK-cell surface marker assay of the VEXAS patient**

| **Cell Subsets** | **Value**  **07/27/2023** | **Value**  **03/18/2024** | **Reference range** |
| --- | --- | --- | --- |
| T lymphocytes (CD3^+^) | 72.36% | 77.73%  (933/μL) | 54.24-83.06%  (711-2353/μL) |
| CD3^+^CD4^+^ | 51.28% | 43.11%  (517/μL) | 22.54-62.84%  (368-1632/μL) |
| CD3^+^CD8^+^ | 17.90% | 27.95%  (335/μL) | 13.28-39.0  (201-931/μL) |
| Th/Ts (CD3^+^CD4^+^/CD3^+^CD8^+^) | 2.86 | 1.54 | 0.53-2.31 |
| B lymphocytes  (CD3^-^CD19^+^) | 20.78% | 12.69%  (152/μL) | 4.12-19.14  (74-534/μL) |
| NK cells  (CD3^-^CD56^+^) | 4.04% | 8.29%  (99/μL) | 4.04-30.86  (63-1013/μL) |

**Supplementary Table 4. Comparison of VEXAS cases complicated with HLH**

|  | | ***This study** | **Grey et al. 2021 ^6^** | ****Kao et al. 2022 ^7^** | **Staels et al. 2021 ^9^** | **Reference range** |
| --- | --- | --- | --- | --- | --- | --- |
| Gender; Age; Nation | | Male; 50; Chinese | Male; 74; Australian | Male; 50; American | Male; 69; European |  |
| *UBA1* mutation | | p.M41V | p.M41T | p.M41T | p.M41T |  |
| Cutaneous involvement | | Erythema nodosum; Maculopapular rash | Neutrophilic  dermatosis | Cutaneous polyarteritis nodosa; Tender cutaneous nodules | Urticarial lesion |  |
| Chondritis | | (+) | (+) | (-) | (+) |  |
| Bone marrow biopsy | | Vacuolated myeloid precursors; Hemophagocytosis | Vacuolated granulocyte precursors | Vacuolated granulocyte precursors; Marked hypercellularity; Hemophagocytosis | Trilineage dysplasia with vacuolization; Hemophagocytosis (CD68^+^) |  |
| Chest CT | | Pulmonary consolidations; GGOs | Pulmonary consolidations; Pulmonary embolus; GGOs | Pulmonary consolidations; Lobe pulmonary nodule |  |  |
| Pathogens | Bacteria | *Haemophilus parainfluenzae, Streptococcus mitis* | *Campylobacter jejuni* | *Actinomyces odontolyticus, Pseudomonas aeruginosa* | Opportunistic  infections; specific pathogens undetermined |  |
|  | Mycobacteria | - | - | - |  |  |
|  | Fungi | *Malassezia furfur* | - | - |  |  |
|  | Virus | *SARS-CoV-2* | - | *EBV* (509,242 copies/mL) |  |  |
|  | Others | *Rickettsia* | N.D. | N.D. |  |  |
| HLH diagnostics criteria | Fever | (+) | (+) | (+) | (+) |  |
|  | Splenomegaly | (-) | (+) | (+); mild | (+) |  |
|  | Hemoglobin (g/L) | 47.00-100.00 (↓) | fall to 66.00 from baseline values of 80.00-90.00 | 67.00 (↓) | 66.00 (↓) | [130-175] |
|  | Platelets (×10^9^/L) | 15.00-210.00 (↓) | acute fall to a nadir of 10 from baseline values of 110-150 | 13.00 (↓) | (↓) | [125-350] |
|  | Triglycerides | 0.78-1.98 mM | >3 mM (↑) | 35.50 mg/L (↑) | (↑) | [<1.7 mM; <15 mg/L] |
|  | Fibrinogen (g/L) | 5.9-18.7 | N.D. | N.D. | N.D. | [2-4] |
|  | Serum ferritin  (ng/mL) | 958.20-3076.00 (↑) | (↑) peaking at 77,232 | 3700-14,198 (↑) | 720.00 (↑) | [30-400] |
|  | sCD25/IL-2R | 2872.76-9880.00 pg/mL (↑) | 41,472 pg/mL (↑) | 6427.00 U/mL (↑) | N.D. | [458-1997 pg/mL; <2400 U/mL] |
|  | NK cell activity (%) | 15.86-16.82 | (↓) | N.D. | N.D. | [>15.11] |
| Serum cytokines | IFN-α (pg/mL) | 39.88 (↑) |  |  |  | [<16.5] |
|  | IFN-γ (pg/mL) | 189.40 (↑) |  | 3.30 |  | [<23.1] |
|  | IL-1β (pg/mL) |  |  | 0.50 |  | [<5.0] |
|  | IL-2 (pg/mL) | 33.04 (↑) |  |  |  | [<7.5] |
|  | IL-5 (pg/mL) | 37.18 (↑) |  |  |  | [<3.1] |
|  | IL-4 (pg/mL) | 10.03 (↑) |  |  |  | [<8.56] |
|  | IL-6 (pg/mL) | 157.26 (↑) | >1000 (↑) | 24.80 (↑) | 102.00 (↑) | [<5.4] |
|  | IL-8 (pg/mL) | 135.12 (↑) | >1000 (↑) | 161.00 (↑) |  | [<20.6] |
|  | IL-10 (pg/mL) | 11.54 | 10,032 (↑) | 48.80 (↑) |  | [<12.9] |
|  | IL-12p70 (pg/mL) | 6.16 (↑) |  |  |  | [<3.4] |
|  | IL-17 (pg/mL) | 49.35 (↑) |  |  |  | [<21.4] |
|  | IL-18 (pg/mL) |  |  | 2627.00 (↑) |  | [<470.0] |
|  | TNF-α (pg/mL) | 0.65 | >300 (↑) | 85.40 (↑) |  | [<16.5] |
| Treatment | | Ruxolitinib was discontinued due to worsened anemia; Tocilizumab (480 mg/3~4 weeks) and a low dose of prednisone (10 mg/d) were regularly given | Refractory to immunosuppression except high doses of glucocorticoids; Treated with pulsed methylprednisone | Rituximab  Methylprednisolone  Dexamethasone  Ruxolitinib | Prednisone  Siltuximab  Rituximab ***  Sirolimus |  |
| Outcome | | Followed up for 8 months till Dec 2024;  No instances of fever;  Hb and PLT levels were maintained at ~70.0-80.0 g/L and ~60.0×10^9^/L respectively | Briefly improved but poor functional status precluded further treatment; Died shortly thereafter | Died from bacterial septic shock due to significant immunosuppression | Resolution of inflammatory symptoms but does not recover trilineage dysplasia |  |

* Clinical data of serum cytokines prior to ruxolitinib treatment (08/15/2023; during the 2nd hospitalization); ** Clinical data from hospitalization prior to treatment; *** no clinical response; CT, computed tomography; GGO, ground-glass opacity; N.D. not determined

**Supplementary Table 5. Results of IFN-γ release assays (IGRA) of the VEXAS patient**

| **Date** | **Value** |
| --- | --- |
| 11/30/2022 | Negative |
| 05/10/2023 | Negative |
| 03/15/2024 | Negative |

**Supplementary Table 6. PCR testings of *cytomegalovirus* (*CMV*) and *Epstein-Barr virus* (*EBV*) of the VEXAS patient**

| **Date** | ***CMV*** | ***EBV*** |
| --- | --- | --- |
| 05/17/2023 | Below the detection limit | 5.08E+02 |
| 07/28/2023 | Below the detection limit | 1.15E+04 |
| 08/07/2023 | Below the detection limit | 3.15E+03 |
| 08/16/2023 | Below the detection limit | 7.18E+03 |
| 08/21/2023 | Below the detection limit | 5.41E+03 |
| 03/15/2024 | Below the detection limit | 4.43E+04 |

**Supplementary Table 7. Coagulation tests of the VEXAS patient**

| **Index** | **Value**  **11/14/2024** | **Value**  **12/05/2024** | **Units** | **Reference range** |
| --- | --- | --- | --- | --- |
| Prothrombin time (PT) | 12.91 | 13.49 | S | [9.2-15.0] |
| Activated partial thromboplastin time (APTT) | 26.75 | 30.28 | S | [21-37] |
| Thrombin time (TT) | 12.77 | 13.16 | S | [10-20] |
| International normalized ratio (PT-INR) | 1.08 | 1.13 |  | [0.8-1.25] |
| Prothrombin time activity (PTA) | 88.02 | 83.92 | % | [70-130] |
| FIB | 5.62 | 6.65 | g/L | [2-4] |
| DD | 0.88 | 0.69 | mg/L | [<0.5] |

**Supplementary Table 8. Other suspected variants in the VEXAS patient detected by the WES analysis**

| Gene | Chr | Variant | VAF | ClinVar | OMIM | Inherit | Type |
| --- | --- | --- | --- | --- | --- | --- | --- |
| *POGZ* | chr1:  151400305 | NM_015100.4:  c.1072A>G | 1.31E-4 | 560580 | White-Sutton syndrome [MIM:616364] | AD | Heter |
| *MYCN* | chr2:  16082889 | NM_005378.6:  c.703G>C | NA | NA | Feingold syndrome-1 [MIM:164280] | AD | Heter |
| *CASP10* | chr2:  202050726 | NM_032977.4:  c.226C>A | 5.44E-5 | 577661 | Autoimmune lymphoproliferative syndrome type IIA [MIM:603909];  Gastric cancer [MIM:613659];  Familial non-Hodgkin lymphoma [MIM:605027] | AD | Heter |
| *KIF1A* | chr2:  241660460 | NM_004321.7:  c.4441-5C>T | 2.97E-3 | 335259 | NESCAV syndrome [MIM:614255];  Hereditary sensory neuropathy type IIC [MIM:614213];  Spastic paraplegia-30A [MIM:610357] | AD/  AR | Heter |
| *IGF2R* | chr6:  160494419 | NM_000876.3: c.4865A>G | 4.89E-4 | NA | Hepatocellular carcinoma [MIM:114550] | NA | Heter |
| *CARD11* | chr7:  2951838 | NM_032415.6:  c.3112G>A | 5.44E-4 | 1383779 | B-cell expansion with NFKB and T-cell anergy [MIM:616452];  Immunodeficiency-11B with atopic dermatitis [MIM:617638];  Immunodeficiency-11A [MIM:615206] | AD/  AR | Heter |
| *SMO* | chr7:  128852104 | NM_005631.5:  c.2176C>T | 3.01E-5 | NA | Pallister-Hall-like syndrome [MIM:241800];  Basal cell carcinoma, susceptibility to, 1 [MIM:605462];  Curry-Jones syndrome [MIM:601707] | AR | Heter |
| *GATA4* | chr8:  11615892 | NM_002052.5:  c.1237C>A | 1.09E-4 | NA | Tetralogy of Fallot [MIM:187500];  Atrial septal defect 2 [MIM:607941];  Ventricular septal defect 1 [MIM:614429];  Atrioventricular septal defect 4 [MIM:614430];  Testicular anomalies with or without congenital heart disease [MIM:615542] | AD | Heter |
| *RAD21* | chr8:  117862904 | NM_006265.3:  c.1573A>G | NA | NA | Cornelia de Lange syndrome 4 [MIM:614701];  Mungan syndrome [MIM:611376] | AD/  AR | Heter |
| *PLEC* | chr8:  144998335 | NM_000445.5:  c.5843G>A | 3.38E-4 | NA | Epidermolysis bullosa simplex 5D [MIM:616487];  Epidermolysis bullosa simplex 5B [MIM:226670];  Epidermolysis bullosa simplex 5C [MIM:612138];  Epidermolysis bullosa simplex 5A [MIM:131950];  Muscular dystrophy, limb-girdle, autosomal recessive 17 [MIM:613723] | AD/  AR | Heter |
| *GRHPR* | chr9:  37432134_37432135 | NM_012203.2:  c.864_865del | 4.89E-4 | 162020 | Hyperoxaluria, primary, type II [MIM:260000] | AR | Heter |
| *EHMT1* | chr9:  140638544 | NM_024757.5:c.1170+2T>C | 5.44E-5 | 2196151 | Kleefstra syndrome 1 [MIM:610253] | AD | Heter |
| *CDON* | chr11:  125831787 | NM_016952.4:  c.3463G>T | NA | NA | Holoprosencephaly 11 [MIM:614226] | AD | Heter |
| *BRCA2* | chr13:  32912670 | NM_000059.3:  c.4178C>T | 6.38E-5 | 89047 | Fanconi anemia, complementation group D1 [MIM:605724];  Glioblastoma 3 [MIM:613029];  Medulloblastoma [MIM:155255];  Prostate cancer [MIM:176807];  Familial breast-ovarian cancer-2 [MIM:612555];  Breast cancer [MIM:114480];  Pancreatic cancer 2 [MIM:613347];  Wilms tumor, type 1 [MIM:194070] | AD/  AR/  SMu | Heter |
| *NBEA* | chr13:  36229860 | NM_015678.4: c.8273A>G | NA | NA | Neurodevelopmental disorder with or without early-onset generalized epilepsy [MIM:619157] | AD | Heter |
| *TINF2* | chr14:  24710891 | NM_001099274.3:c.389C>T | 1.68E-4 | 1004809 | Dyskeratosis congenita, autosomal dominant 3 [MIM:613990];  Revesz syndrome [MIM:268130] | AD | Heter |
| *TBX6* | chr16:  30097630 | NM_004608.3:  c.1227G>A | 4.56E-1 | 259448 | Spondylocostal dysostosis 5 [MIM:122600] | AD/  AR | Heter |
| *KRT10* | chr17:  38975103  _  38975104 | NM_000421.4:c.1683_1684insAGCTCCGGCGGCGGATACGGCGGCGGCAGCAGCTCCGGCGGCGGATACGGCGGCGGCAGC | NA | 1049274 | Ichthyosis, annular epidermolytic 1 [MIM:607602];  Epidermolytic hyperkeratosis 1 [MIM:113800];  Ichthyosis histrix, Lambert type [MIM:146600];  Ichthyosis with confetti [MIM:609165] | AD/  AR | Heter |
| *CACNA1G* | chr17:  48701816 | NM_018896.5:  c.6325C>G | 3.59E-4 | NA | Spinocerebellar ataxia 42 [MIM:616795];  Spinocerebellar ataxia 42, early-onset, severe, with neurodevelopmental deficits [MIM:618087] | AD | Heter |
| *INSR* | chr19:  7117188 | NM_000208.4:  c.4028G>A | 4.35E-4 | 330439 | Rabson-Mendenhall syndrome [MIM:262190];  Diabetes mellitus, insulin-resistant, with acanthosis nigricans [MIM:610549];  Donohue syndrome [MIM:246200];  familial hyperinsulinemic hypoglycemia-5 [MIM:609968] | AD/  AR | Heter |
| *TRPM4* | chr19:  49674888_49674889 | NM_017636.4:  c.912_913del | 5.45E-5 | 942166 | Progressive familial heart block type IB [MIM:604559];  Erythrokeratodermia variabilis et progressiva-6 [MIM:618531] | AD | Heter |
| *MYH9* | chr22: 36684817 | NM_002473.5:  c.4726C>T | 3.59E-4 | NA | Macrothrombocytopenia and granulocyte inclusions with or without nephritis or sensorineural hearing loss [MIM:155100];  Autosomal dominant deafness-17 [MIM:603622] | AD | Heter |
| *ATRX* | chrX:  76763876 | NM_000489.5:  c.7432C>G | 5.05E-4 | 521610 | X-linked alpha-thalassemia/impaired intellectual development syndrome [MIM:301040];  Alpha-thalassemia myelodysplasia syndrome [MIM:300448];  X-linked intellectual disability-hypotonic facies syndrome-1 [MIM:309580] | XLD/  XLR | Hemi |
| *ATP7A* | chrX:  77294454 | NM_000052.7:  c.3632G>A | 2.62E-4 | 590222 | Occipital horn syndrome [MIM:304150];  X-linked distal hereditary motor neuronopathy [MIM:300489];  Menkes disease [MIM:309400] | XLR | Hemi |

***Materials and Methods***

Subjects

The patient was enrolled in the Xiangya Hospital of Central South University, Hunan, China. Two vials of peripheral blood from him and his family members were collected, respectively, in EDTA containing vacutainer tubes. One vial of blood samples was sent for WES analysis (KingMed Diagnostics, China), and the other vial was used for PCR and sanger sequencing.

Genomic DNA and PCR

Blood samples were spin down at 500 g for 5 min. The cell pellets were digested with 6 volumes of red blood cell lysis buffer (Beyotime Biotech #C3702, Shanghai, China) for 5 min. The samples were spin down again to collect cell pellets. Digest cell pellets with DNA lysis buffer supplemented with 0.5 mg/ml Proteinase K at a 55℃ waterbath for overnight. We then employed the method of standard phenol/chloroform to obtain the genomic DNAs. DNAs were then amplified by genomic PCR using the UBA1 primers (UBA1-F: ACCCATGTGCTCCAGGGTC; UBA1-R: AGGACACTGGATGTCTGGAGC), and run on a 1.5% agarose gel. The correct bands (500 bp) were cut out and sanger-sequenced by the UBA1-F primer.

***Full Case Descriptions***

The brief medical history is summarized in a chronicle in ***Figure S2***. The patient, a 50 year-old man, was admitted to our department on Mar 13, 2024, for recurrent cutaneous erythema nodosum for more than 1 year and recurrent fever for 10 months. The patient had a past history of chronic viral hepatitis B for more than 10 years. Since Aug 2022, the patient had maculopapular rash on both lower limbs, which were scattered and raised on the skin, accompanied by pain and itching. Erythema on scalp with pain and erythema on external auricles appeared in the following 2 months, he was treated with methylprednisolone (starting at 40 mg/d and tapering) in other hospital, and the above symptoms improved. The patient visited our outpatient department in Nov 2022, and was initially diagnosed with relapsing polychondritis (RP), and was treated with prednisone (15 mg/d), methotrexate (10 mg/week), iguratimod to suppress inflammation, and entecavir to prevent HBV replication. The patient's rash and auricular erythema were improved, and prednisone were gradually reduced to 5 mg/d.

In Dec 2022, the patient presented with fever, cough, chills, and muscle aches and pains, and visited the emergency department of our hospital. The laboratory tests showed mild anemia, and the nucleic acid test for the COVID-19 was positive. CT images manifested multiple flaky, ground-glass opacities (GGOs) and striated hyperdensities with blurred margins in both lungs. Solid shadows were seen in the lower lobes of both lungs with tracheal signs visible within. His temperature returned to normal after being treated with azulfidine antiviral and symptomatic therapy. Since then, he was regularly revisited in our outpatient department every month. Laboratory tests showed mild anemia and gradual reduction of platelets (PLT). Inflammatory markers, such as C-reactive protein (CRP), erythrocyte sedimentation rate (ESR), and serum ferritin were all significantly elevated. In Apr 2023, due to the relapse of auricular erythema, the prednisone dosage was again increased to 10 mg/d.

The patient was admitted to the department of respiratory medicine of our hospital in May 2023 due to fever, cough, sputum and shortness of breath. On May 6, chest CT showed lung infections. The galactomannan (GM) test, automatic smear antacid staining test, Xpert MTB/RIF assay, as well as aerobic culture and smear of sputum, were all negative. There was no obvious improvement after empiric antibiotic therapy. Further next-generation sequencing (NGS) testing of bronchoalveolar lavage fluid (BALF) suggested rickettsial infection, and doxycycline was given.

However, the patient still had recurrent fever, and his degree of anemia and PLT counts were significantly reduced. Bone marrow aspiration was performed, which revealed hypercellular bone marrow, increased ratio of myeloid lineage with cytoplasm vacuoles and toxic granules, decreased ratio of erythroid cells, presence of naive lymphocytes (1%), reticulocytes and hemophagocytosis, hindered megakaryocyte maturation and sparse distribution of PLT. The blood smear showed increases in lobulated nuclei of neutrophils, presence of cytoplasmic vacuoles in myeloid precursors, as well as sparse PLT distribution. The activity of peripheral natural killer (NK) cells was 15.86% (reference value ≥15.11), and the [plasma levels of soluble interleukin-2 receptor α (](https://www.ahajournals.org/doi/10.1161/ATVBAHA.115.305289)sCD25/IL-2Rα) was 2872.76 pg/mL (reference value 458-1997 pg/mL).

Because the patient had recurrent fever, hemocytopenia, elevated serum ferritin, hemophagocytosis in bone marrow and elevated sCD25/IL-2Rα, concomitant of infection-associated hemophagocytic syndrome was considered. The patient was then treated with methylprednisolone (40 mg/d) and transfused with 2 U of concentrated red blood cells (RBCs) to improve anemia symptoms. Upon discharge on Jun 2, 2023, his temperature returned to normal, so the corticosteroid was reduced to prednisone (15 mg/day, orally), and continuation of doxycycline for 7 days was recommended.

About 5 days after discharge, the patient developed red maculopapular rashes on the trunk and extremities without itching, followed by fever. He was then hospitalized in other hospital from Jun 12 to Jun 25, 2023, during which the rash gradually worsened. Targeted NGS (tNGS) analysis of BALF was performed, and no evidence of pathogens was detected. The patient was treated with methylprednisolone via intravenous (iv) , the symptoms of fever and rash were improved and later he was discharged.

He continued to take methylprednisolone (20 mg/d). Since July 12, 2023, the patient, again, had fever every day, with a maximum temperature of 40 ℃. On July 20, 2023, he present to the emergency department of our hospital and performed a chest CT examination, compared with CT images of Jun 2023, there were new infectious lesions in both lungs and increased lesions in the lower lobe of the left lung. Degree of anemia and thrombocytopenia worsened again (hemoglobin (Hb) level was 54 g/L; PLT count was 35×10^9^/L). In addition, serum ferritin level was 2034 ng/mL and the level of sCD25/IL-2Rα was 9880 pg/mL, both were far above normal ranges. Moreover, the NK cell activity was 16.82%. Plasma fibrinogen was 9.01 g/L and IL-6 level was 68.2 pg/mL. Blood cultures (bacterial, fungal, anaerobic) were negative.

The 18F-fluorodeoxyglucose positron emission tomography (PET/CT) demonstrating hypermetabolic activity in bone marrow and no signs of malignant solid tumors in the systemic detection sites (**Figure S1**). The patient was treated initially with antibiotic, such as piperacillin/tazobactam (July 20-23, 2023), meropenem (July 23-26, 2023). Then dexamethasone (10 mg/d) was given to repress recurred fever which was considered due to atypical phagocytosis syndrome. On July 26, 2023, he was admitted to the department of hematology of our hospital. Further tests of the blood culture, sputum culture, and bone marrow culture were negative. The findings of bone marrow aspiration were roughly the same as before. The flow cytometry of bone marrow lymphocytes showed no significant clonal B cells or clonal plasma cells, and also there were no significant abnormalities in T lymphocyte phenotype. Chromosomal karyotype analysis showed no significant abnormalities. Hence, all those results suggest no evidence of myelodysplastic syndrome (MDS). The antacid staining and the Xpert MTB/RIF assay of BALF were all negative. NGS analysis of BALF revealed *Haemophilus parainfluenzae* (sequence number 51, relative abundance 0.502%), *Streptococcus mitis* (18, relative abundance 0.177%), and *Malassezia furfur* (sequence number 40, relative abundance 1.059%). DNA test of *Epstein-Barr virus* (*EBV*) in peripheral blood was 1.15×10^4^ copies/mL. However, nucleic acid quantification in sorted peripheral blood single nucleated cells showed that *EBV* load was 2.03×10^2^ copies/mL. In sorted B cells, *EBV* load was 14.25 copies/10^5^ cells, suggesting that *EBV* was latent in peripheral blood.

Treatment was continued with dexamethasone (10 mg/d), cefoperazone sulbactam anti-bacterial, and caspofungin anti-fungal. The patient's temperature continued to be normal. Dexamethasone was thus reduced to 5 mg qd on Aug 2, 2023, after which the patient developed recurrent fever. A wide spectrum of inflammatory factors as detected were elevated markedly (**Table S2**). Thus, ruxolitinib (5 mg twice daily) was then added on Aug 16 to control inflammation. Five days after ruxolitinib treatment (08/21/2023), serum cytokines such as IFN-α, IFN-γ, IL-2, IL-5, IL-8, IL-12p70 and IL-17 were remarkably reduced (**Table S2**), but he still had fever and cough. A repeat chest CT on Aug 19 showed that the infectious lesions in the upper lobes of both lungs were slightly reduced after the previous treatment, and the lesions in the lower lobes of both lungs were roughly the same as before. He was discharged on August 22, 2023 and is on regular ruxolitinib and dexamethasone (1.5 mg/day).

From Nov 2023 to Mar 2024, the patient was repeatedly seen in other hospitals, because of fever. Whole-exome sequencing (WES) (KingMed Diagnostics, Guangzhou, China) was performed on Nov 21, 2023, which revealed a hemizygous NM_003334.4:c.121A>G (p.M41V) *UBA1* mutation in this patient at a variant allele frequency (VAF) of 69.6%. The diagnosis was then made with VEXAS syndrome. During that period, meropenem, piperacillin/tazobactam anti-bacterial infection, fluconazole anti-fungal infection, methylprednisolone sodium succinate (40 mg; iv) and blood transfusion, as well as other symptomatic treatments were given. **Ruxolitinib was discontinued in Feb 2024 due to worsened anemia (no specific tests were reported).** Tocilizumab (162 mg subcutaneous injection once) was then given on Feb 29, 2024. The patient's febrile symptoms were greatly improved. After discharge from the hospital, the patient was regularly treated with methylprednisolone (8 mg/d) and fluconazole (200 mg/d). On Mar 8, 2024, fever appeared again, with a maximum temperature of 38.5°C, accompanied by profuse sweating. The temperature could return to normal after sweating, accompanied by a new rash. He was hospitalized in our department of rheumatology and immunology, Xiangya Hospital, on Mar 13, 2024.

After the admission, physical examinations showed: Temperature (37.1 ℃); Pulse rate (103 times/min); Respiratory rate (20 times/min); Blood pressure (99/64 mmHg); abdominal obesity; facial rounding and plethora (“moon face”); supraclavicular and dorsocervical fat pads (“buffalo hump”). The whole body can be seen gray scaly patches. Head, face, trunk and limbs can be seen in the new onset of bright red maculopapular rashes, some of which with the subcutaneous nodules and pressure pain. The skin has no hemorrhagic spots or bruises. Superficial lymph nodes were not enlarged. The conjunctiva and lips were pale. Bilateral auricles were red and swollen without tenderness. The bridge of the nose was not collapsed. Breath sounds were normal, but wet rales could be heard in both lower lungs.

On Mar 14, 2024, blood tests showed RBC count (1.81 × 10^12^/L), Hb (67.0 g/L), PLT count (62 × 10^9^/L), mean corpuscular volume (MCV; 110.1 fl), serum ferritin (3076.0 ng/mL). Serum markers, including CRP, ESR, procalcitonin (PCT), D-dimer (DD), triglycerides (TG) and fibrinogen (FIB), were tested (**Table S1**). After tocilizumab treatment, tests of serum cytokines showed that IL-6 and IFN-γ level was 298.25 pg/mL and 130.82 pg/mL, respectively, while levels of other cytokines such as IFN-α, IL-2, IL-4, IL-5, IL-8, IL-12p70 and IL-17 were all decreased back to normal ranges (**Table S2**). Immunoglobin (IgA, IgG, IgM) and complement (C3, C4) levels, and the lymphocytes, assessed by TBNK assays, were all within normal ranges (**Table S3**). Tuberculosis was not detected by IFN-γ release assays (IGRA)(**Table S5**). C12 tumor markers and thyroid function were also normal. *Cytomegalovirus (CMV)* and *EBV* were inactivated (**Table S6**).

Compared with CT images of Aug 2023, chest CT showed that multiple foci of infection in both lungs were absorbed. Color doppler ultrasound (CDU) showed diffuse lesions in liver parenchyma, gallbladder stones, polypoid lesions of gallbladder, cholecystitis, multiple stones in both kidneys, and prostatic hyperplasia with multiple foci of calcification. Echocardiogram (ECG) showed slight left atrial enlargement, widening pulmonary artery, mild regurgitation of the bicuspid and tricuspid valves and pulmonary valve, and ventricular compliance hypoplasia (heart rate: 107 beats/minute).

The patient was treated with prednisolone (10 mg/d), anti-hepatitis B virus drugs, and other symptomatic supportive treatments. Considering that the patient's fever was related to VEXAS syndrome, he was treated with tocilizumab (480 mg/4 weeks; iv). After that, the symptoms of fever and rash were ameliorated. Moreover, the PLT count was elevated (73.0×10^9^/L), and the levels of CRP and serum ferritin were going lower.

On the morning of Mar 19, 2024, the patient had sudden dyspnea with cough, and chest pains. His finger pulse oxygen saturation fluctuated between 60%-80%. He was immediately given nasal catheter oxygen and then changed to transnasal high-flow oxygen. Arterial blood gas analysis showed pO_2_ (67.0 mmHg), Glu (6.5 mmol/L), Lac (3.5 mmol/L), pO_2_(T) (65.0 mmHg pH 7.45), HCO_3_^-^ (27.1 mmol/L) and FiO_2_ (70.0%). The oxygenation index was 96. Chest CT showed new multiple infections in both lungs, several with consolidation changes. Given the patient's underlying disease and immunosuppressive status, as well as a history of close contact with *SARS-CoV-2* virus, it was considered that pneumonia might be due to COVID-19. The patient was thus treated with a combination of antiviral therapy of simnotrelvir/ritonavir tablets.

On Mar 20, 2024, the COVID-19 was, however, tested negative. The patient was then treated with anti-bacterial levofloxacin sodium chloride (0.5 g qd; iv), continued antifungal fluconazole (200 mg qd; orally), and anti-inflammatory methylprednisolone sodium succinate (40 mg qd; iv). After that, the patient’s hypoxia symptoms improved with blood oxygen saturation maintained at more than 95%. Arterial blood gas analysis showed pO_2_ (112.0 mmHg), Ca^2+^ (1.47 mmol/L), Glu (6.9 mmol/L), Lac (4.4 mmol/L), pO_2_ (T) (106.0 mmHg) and FiO_2_ (60.0%). The oxygenation index was 186. Blood tests showed leukocyte count (15.7×10^9^/L), Hb (61.0 g/L), PLT count (67.0×10^9^/L; decreased than before). The serum ferritin level was 2130.0 ng/mL.

On Mar 21, 2024, levofloxacin was discontinued and replaced by meropenem (1 g q8h iv) to strengthen the anti-infection. Considering the patient's high D-dimer levels and the hypercoagulable state of the blood upon inflammation (**Table S1**), he was given subcutaneous injections of low molecular heparin (4000 U) to prevent thrombosis for 3 days. After that, the patient's symptoms of shortness of breath, cough, and chest pains were significantly improved. The blood test was then performed and showed that PLT count decreased to 37.0×10^9^/L and CRP level was reduced to 6.84 mg/L. Blood cultures (bacterial, fungal, anaerobic) were negative. The chest CT showed that multiple infections in both lungs were absorbed compared with the previous one. On Mar 25, 2024, the blood test was again performed and showed RBC count (1.67×10^12^/L), Hb (61.0 g/L) and PLT count (21.0×10^9^/L). The CRP level was back to the normal range.

On Mar 27, 2024, arterial blood gas analysis showed that the oxygenation index was 467 (normal range). The blood test showed RBC count (1.68×10^12^/L), Hb (61.0 g/L) and PLT count (34.0×10^9^/L). Given that the patient was prone to recurrent infections due to immunosuppressive therapies, compound sulfamethoxazole tablets (0.48 g orally every other day) were added to prevent *Pneumocystis jiroveci* pneumonia (PJP). Overall, after the combined treatments, the patient’s rash and cough symptoms were greatly improved. He was discharged on Mar 28, 2024 without fevers and dyspnea.

After that, tocilizumab (480 mg/3~4 weeks) and a low dose of prednisone (10 mg/d and tapering to 7.5 mg/d) were regularly given. The patient was then followed up for a period of 8 months until Dec. 2024, during which there were no instances of fever. Initially, before receiving tocilizumab injections, the patient experienced mild muscle aches/pains or a few new rashes, along with elevated levels of CRP (***Table S1***). However, all of these symptoms could be alleviated after the administration of tocilizumab. Basically, the Hb level was maintained at around 70.0~80.0 g/L, and the PLT level was maintained at around 60.0×10^9^/L (**Fig. 1**). The coagulation examination was relatively normal (**Table S7**). As such, the patient was in a stable condition that greatly improved his life quality.
